# Acoustic-propelled micro/nanomotors and nanoparticles for biomedical research, diagnosis, and therapeutic applications

**DOI:** 10.3389/fbioe.2023.1276485

**Published:** 2023-10-19

**Authors:** Guanyu Mu, Yu Qiao, Mingyang Sui, Kenneth T. V. Grattan, Huijuan Dong, Jie Zhao

**Affiliations:** ^1^ State Key Laboratory of Robotics and System, Harbin Institute of Technology, Harbin, China; ^2^ School of Science and Technology, University of London, London, United Kingdom

**Keywords:** micro/nanomotors, targeted drug delivery, biocompatibility, acoustic manipulation, biomedical engineering

## Abstract

Acoustic manipulation techniques have gained significant attention across various fields, particularly in medical diagnosis and biochemical research, due to their biocompatibility and non-contact operation. In this article, we review the broad range of biomedical applications of micro/nano-motors that use acoustic manipulation methods, with a specific focus on cell manipulation, targeted drug release for cancer treatment and genetic disease diagnosis. These applications are facilitated by acoustic-propelled micro/nano-motors and nanoparticles which are manipulated by acoustic tweezers. Acoustic systems enable high precision positioning and can be effectively combined with magnetic manipulation techniques. Furthermore, acoustic propulsion facilitates faster transportation speeds, making it suitable for tasks in blood flow, allowing for precise positioning and in-body manipulation of cells, microprobes, and drugs. By summarizing and understanding these acoustic manipulation methods, this review aims to provide a summary and discussion of the acoustic manipulation methods for biomedical research, diagnostic, and therapeutic applications.

## 1 Introduction

In the past decade, various methods have been used to manipulate micro droplets and particles, including electrodynamics (Pollack et al., 2000; Li and Kim, 2020), acoustics (Wiklund et al., 2012; Zhang et al., 2020), electromagnetics (Fan et al., 2020), and fluid mechanics (Zhu and Wang, 2017). Acoustic levitation and manipulation are achieved by adjusting the position of trapping point through active adjustment of the acoustic field, thus there are no specific requirements for the shape or attributes of the manipulated objects compared with other transportation methods (Li et al., 2018; Wang et al., 2022; Zhang et al., 2023). Therefore, acoustic methods has shown a number of important benefits in biomedical applications: such as simple configuration and low cost, high biocompatibility and low contamination of reagents (Zhang et al., 2018) as well as high-speed fluid driving and large driving force. Due to the above advantages, acoustic manipulation of bio-matter in air or water has demonstrated various applications in medicine (Guttenberg et al., 2005; Zhang et al., 2011; Peng Lee et al., 2013; Schmid et al., 2014) and biological research (Mohanty et al., 2020).

Specifically, the acoustic manipulation can be divided into Bulk Acoustic Wave (BAW) type and Surface Acoustic Wave (SAW) type according to the mode of wave propagation. BAWs are normally excited by PZT materials and they have a wide range of working frequency (from tens of kHz to tens of MHz), meaning that BAWs can manipulate objects ranging in size from millimeters to nanometers, and being particularly suitable for guiding and micro-manipulating inside biological organisms. By comparison, inter-digital transducers (IDT) are often used to generate SAW. Its operating frequency is from several hundred KHz to tens of MHz, and SAW is very ideal for acoustic manipulation of micro- and nano-objects in liquid medium, for the purpose of separation and detection. In this review, the ability of acoustic manipulation for precise delivery is vital in diverse biological research domains, such as intracellular substance delivery and controlled cell growth, targeted drug delivery and genetic marker delivery for diagnostic purpose. This review introduces the three common techniques of acoustic manipulation for micro/nano-motors: traveling waves, standing waves, and phased arrays. Additionally, we provide a summary of the medical applications of acoustic-driven micro/nano-motors.

## 2 Acoustic manipulation techniques commonly used in biological applications

### 2.1 Manipulation technique using standing waves

#### 2.1.1 Operation principle of standing wave manipulation

Standing Wave (SW) acoustic manipulation is a technique that harnesses the power of standing waves to control and manipulate micro-objects such as particles or cells within air or a liquid medium. It operates on the principle of constructive interference between two counter-propagating sound waves. When these waves align, they create stable regions of high and low pressure, known as antinodes and nodes, respectively, within the fluid. Particles suspended in this medium experience acoustic radiation forces that drive them towards the nodes. By adjusting the phase and frequency of the sound waves, precise control over particle positioning can be achieved. SW manipulation finds applications in various fields, including biomedicine, microfluidics, and materials science, enabling tasks like cell sorting, patterning, and precise particle assembly.

**FIGURE 1:**
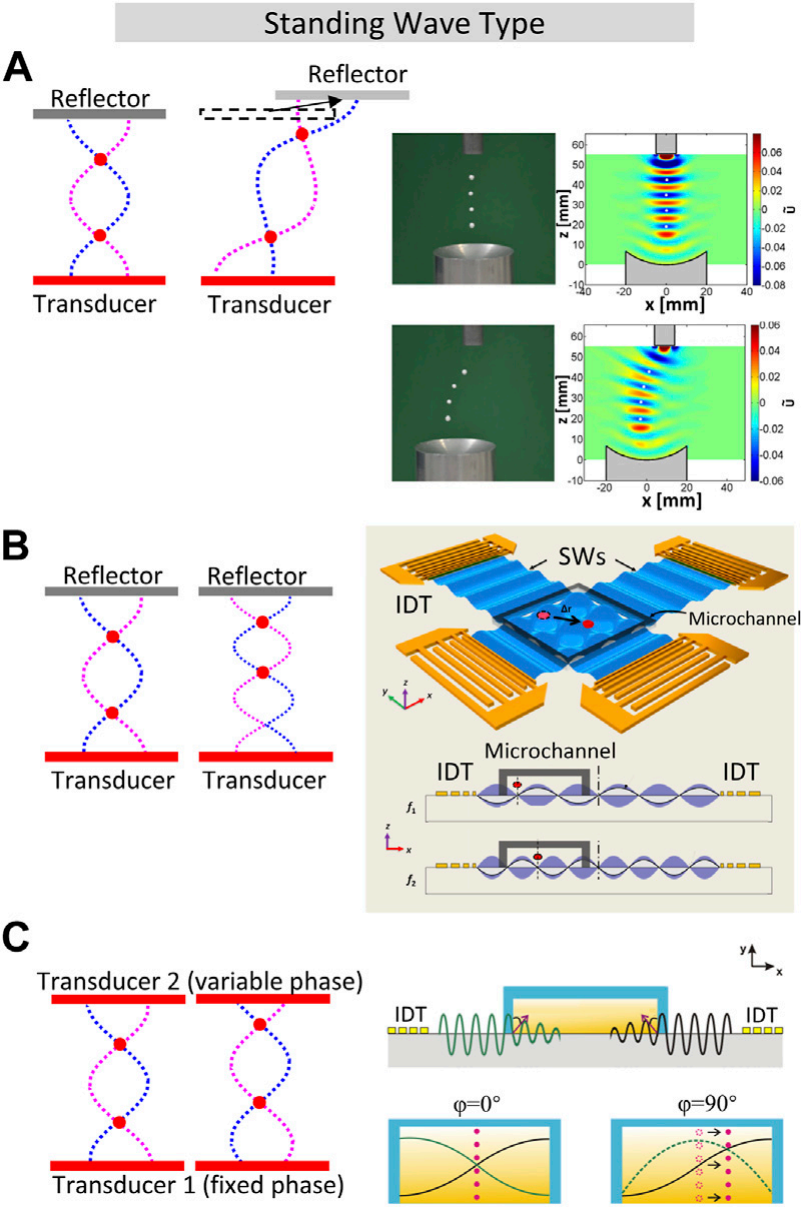
Acoustic manipulation of particles and droplets using standing waves (SW). **(A)**. SW method by moving the reflector–normally used for manipulate macro-objects. Adapted and reproduced with permission from (Andrade et al., 2015). Copyright 2015, AIP Publishing LLC. **(B)**. Standing Surface Acoustic Wave (SSAW) manipulation of biological objects, including cells and entire organisms (*C. elegans*) by adjusting the frequency of two chirped IDTs (Ding et al., 2012). **(C)**. Single human breast cancer cell manipulation using SAW by adjusting the phase shift between opposed IDTs. Reproduced with permission from (Meng et al., 2011). Copyright 2011, American Institute of Physics.

SW is formed between the radiator and the reflector when their distance is an integer multiple of the half wavelength. Therefore, the SW node could be moved by adjusting the structural distance and control the wavelength by changing the frequency at the same time. In particular, (Fletcher et al., 1975), realized the movement of solid objects in the acoustic field through adjusting the position of the movable wall while concurrently modifying the frequency of the opposing speaker to keep the acoustic chamber resonant and as a result, the object was transported half the distance moved by the movable wall. This method of moving the reflector can also realize in-plane manipulation in non-resonant mode (Andrade et al., 2015), as shown in Figure 1A. In this mode, the SW is formed by the superposition of the sound wave emitted by the transducer and the first reflected wave, while higher-order reflections can be ignored because the size of the transducer is relatively small compared to the size of the reflector. Thus, the distance between the excitor and reflector does not have to be an integer multiple of the wavelength anymore. In their actual setup, the diameter of the piezoelectric transducer and the concave reflector is 10 mm and 40 mm, respectively. The polystyrene sphere is levitated at a node about a quarter wavelength away from the transducer surface and its position can be changed by moving the reflector. Due to the requirement of mechanical part to move the reflector, this SW transportation method based on moving the reflector is difficult to apply to SAW devices, which usually has a fixed distance between the transducers. Also, the wavelengths required for micrometer-sized cells/micro/nano-motors are much shorter than in air manipulation of millimeter-sized objects, making it challenging to move the reflector in such a precise way.

#### 2.1.2 Biotweezer using frequency variation method

To manipulate micro/nanoscale objects in liquid, one-dimensional acoustic transportation can be achieved without any moving parts, by switching the frequency between different resonant modes in a resonant cavity with fixed ends.

Ding et al. (Ding et al., 2012) employed two orthogonal pairs of chirped interdigital transducer (IDT) devices to generate SAW and micro and nanoscale biological objects, where *C. elegans* were manipulated through frequency adjustment, as depicted in Figure 1B. Their experiments also ensured that acoustic field is safe to biological samples, because the power density of acoustic tweezers was much lower compared to optical tweezers and optoelectronic tweezers. (Jooss et al., 2022). conducted *in vivo* experiments on the manipulation of microbubble particles in zebrafish embryos. Using orthogonal opposing PZT transducers, they created a high frequency (∼4.0 MHz) BAW field within the zebrafish embryo and adjusted the sound pressure nodes through frequency modulation. As a result, controlled up-, down-, and cross-stream manipulation of the entire vasculature system was achieved. This enabled precise control of microbubbles *in vivo* for biomedical applications.

#### 2.1.3 Biotweezer using phase control approach

Acoustic manipulation by adjusting the phase provides a more continuous and stable one-dimensional levitation transportation capability compared to the above two methods (Park and Ro, 2013). This is because the transducers maintain a fixed excitation frequency throughout the transportation process using the phase control method, thereby allowing the utilization of transducers with high quality factors to enhance the sound pressure of the acoustic field. Consequently, the method of generating SWs through the superposition of traveling waves emitted by a pair of transducers was developed (Matsui et al., 1995; Haake and Dual, 2002; Kozuka et al., 2007; Courtney et al., 2012; Dong et al., 2017). The position of the pressure nodes has a linear relationship with the phase difference between the two transducers. As early as 1995, (Matsui et al., 1995), conducted experiments on the relationship between the phase difference, the levitation position, and the acoustic radiation force in the air using a coaxial dual transducer device. This method is then used for precise transportation of single human breast cancer cell, the benefits of sonoporation in increasing cell permeability are also obtained simultaneously (Meng et al., 2011; Meng et al., 2014), as shown in Figure 1C.

Apart from coaxial dual transducer devices, different forms of resonant cavities could be used to expand the transportation range (Kozuka et al., 2007; Park and Ro, 2013). On a more microscopic scale, (Bernassau et al., 2013), developed an acoustic tweezer using seven PZT transducers. By selectively exciting multiple transducers at 4 MHz and adjusting the phase difference between them, they generated line or hexagonal-shaped acoustic fields for cell patterning. They successfully patterned and cultured C2C12 cell line and Schwann cells, forming columnar structures under the lattice acoustic field, suggesting potential applications in tissue engineering.

### 2.2 Fast transportation of micro/nano-swimmers using traveling waves

During SW transportation, the objects are captured at the SW nodes, which limits the shapes and sizes of objects and restrict the speed and distance of transportation. However, the Traveling Wave (TW) transportation method has overcome these limitations, the TW method involves the generation of acoustic waves that propagate through a medium in a single direction. Unlike SWs, where waves interact to create stationary nodes and antinodes, traveling waves maintain their directional movement. In TW manipulation, these waves create a force field that can be harnessed to control the movement and positioning of micro- and nanoparticles suspended within the medium. This method is particularly advantageous for achieving rapid and precise transport of particles continuously over long distances and travel at high speeds.

**FIGURE 2:**
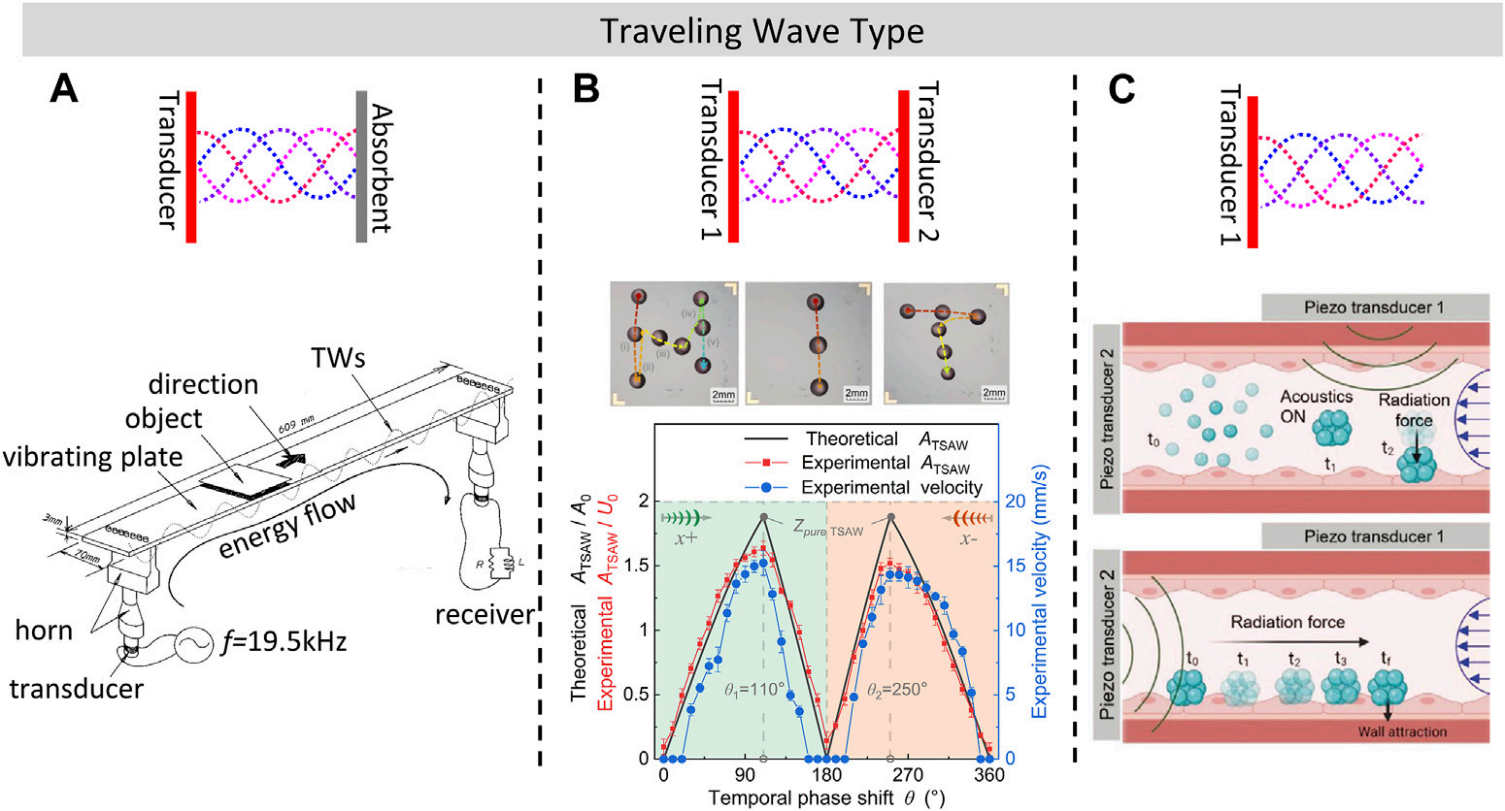
Line transportation and manipulation in plane using TW **(A)**. Acoustic transportation device using TW formed with passive electric network. Reprinted with permission from (Hashimoto et al., 1998). Copyright 1998, Acoustic Society of America. **(B)**. Droplet transportation by adjusting the temporal phase shift of SAW. Reproduced with permission from (Sui et al., 2022). Copyright 2022, The Royal Society of Chemistry. **(C)**. Traveling Surface Acoustic Wave (TSAW) manipulation of Swarmbots inside blood flow using a focused IDT (Fonseca et al., 2022). Copyright 2022 The Authors. Advanced Materials Interfaces published by Wiley-VCH GmbH. This is an open access article under the terms of the Creative Commons Attribution-Noncommercial License.

In air-based TW transportation systems, the “excitation-absorption” mode is commonly used to generate TWs, where either a transducer or absorbent materials can be utilized to absorb vibrations and thus form TWs along the vibrating plate. (Hashimoto et al., 1998; Ueha et al., 2000). achieved TW-based near field acoustic levitation and transportation using two transducers and a vibrating plate, as shown in Figure 2A, where a TW with standing wave ratio of 1.7 was formed. The terminal velocity of a large planar object weighed 8.6 g reached 0.7 m/s, which is significantly faster compared to SW transportation methods. Absorbent materials such as silicone rubber can also be utilized to generate a TW acoustic field in air to transport ethanol droplets (Ito et al., 2010; Ding et al., 2012).

The “excitation-absorption” mode can also be used to generate travelling surface acoustic waves (TSAW), so that liquid-based transportation of micro/nano robots can be achieved. TSAW could be formed either by the use of sound-absorbing gels (Renaudin et al., 2006; Li et al., 2007; Collignon et al., 2015) or by setting a spatial phase difference between the pairs of the IDTs (Sui et al., 2022; Dong et al., 2023), as shown in Figure 2B. Another method for forming TSAW is to use single curved-shape transducer that operates at high excitation frequency from 100 MHz to 1 GHz (Destgeer et al., 2017). Higher frequency TSAW is suitable for precise transportation of micro-scale objects such as selective cell sorting because of its shorter acoustic wavelength (Collins et al., 2016). Using similar principles, (Fonseca et al., 2022), generated TWs in liquid with one PZT transducer to simulate physiological flow environments in blood vessels, as illustrated in Figure 2C. Operations in blood vessels was found to be a complex and challenging procedure (Zhang et al., 2023), while the acoustic method could also successfully transport micro/nanorobots across the flow and upstream without special requirements on the material used compared to magnetic propulsion methods (Li et al., 2023), and it will not generate any additional magnetic fields. As micro/nanorobots have many potential applications in medical field, such as drug delivery (Yu et al., 2023) and noncontact biomedical operations (Ji et al., 2021), and it has been demonstrated (Yu et al., 2022) that passive micro/nanomotors are capable of being moved in complex 3D environments, which enables the possibility to realize targeted drug delivery by generating a localized acoustic field.

### 2.3 Transportation methods based on transducer arrays and holograms

Phased array transducers represent an acoustic manipulation technique that leverages an array of transducers, typically positioned at precise intervals, emit acoustic waves with controllable phase shifts. By carefully adjusting the timing and phase of the emission of each transducer, it is possible to manipulate the resultant acoustic field dynamically. This dynamic control allows for the creation of intricate patterns of pressure nodes and antinodes within the field. Consequently, small objects or particles within this acoustic landscape can be precisely levitated and manipulated in real-time and in three dimensions. The transducer array could realize long-distance transportation of multiple objects by changing the acoustic focal points, and the transportation process is more stable compared to the above-mentioned SW method. By controlling the excitation phase difference and amplitude of the driving voltage applied to adjacent transducers, SW nodes can be generated at any position in space. In 2014, (Hoshi et al., 2014; Ochiai et al., 2014a; b), used two opposite ultrasonic phased arrays to realize three-dimensional transport of millimeter-sized polystyrene particles in air. The range of the sound field can vary widely compared to the SW method and TW method, from 25 × 25 cm^2^ to 100 × 100 cm^2^. The transducer arrays can also realize acoustic focus by adjusting the orientation of the transducers mechanically (Marzo et al., 2017; Youssefi and Diller, 2019; MatthewáReichert, 2020) instead of modulating the phase between the transducers, the advantage is to maximize the acoustic radiation force and reduce parasitic reflections.

**FIGURE 3:**
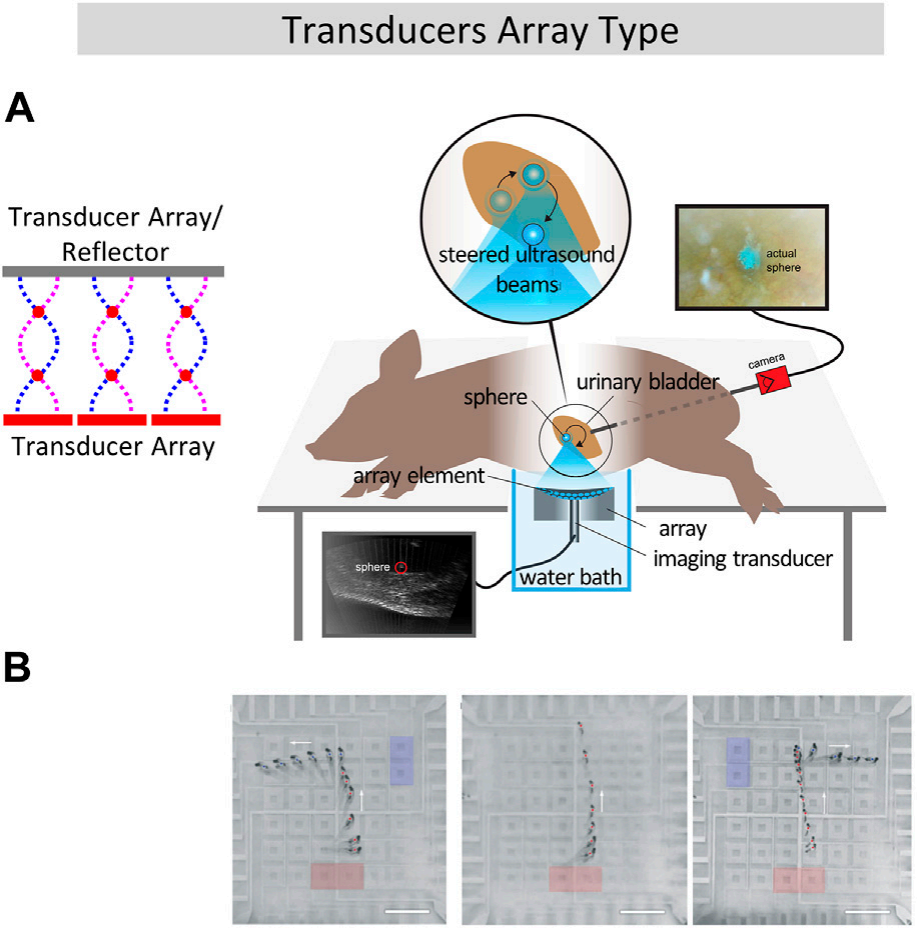
Biomedical application of line transportation using transducer arrays. **(A)** Transcutaneous acoustic manipulation of a 3-mm glass sphere inserted through the ureter into the bladder of a pig using ultrasonic phased array (Ghanem et al., 2020). **(B)** Programmable acoustofluidic manipulation of zebrafish larvae using array of hollowsquare shaped IDTs. Reproduced with permission from (Zhang et al., 2019). Copyright 2019, The Royal Society of Chemistry.

Compared with opposed dual arrays, single-sided array reduces the cost and does not create additional acoustic nodes. In the single-sided array configuration type, (Koyama and Nakamura, 2010; Nakamura and Koyama, 2012; Kashima et al., 2014), built a circular piezoelectric transducer array composed of 24 piezoelectric transducers. By switching the input signal between the piezoelectric patches, manipulation of polystyrene ball with circular trajectory with an accuracy of 7.5° was realized. Daniele et al. (Foresti et al., 2013a; Foresti et al., 2013b; Vasileiou et al., 2016) developed a one-dimensional array composed of multiple 15 × 15 mm transducers. Contactless droplet mixing and cell DNA transfection were achieved. In 2015, Marzo et al. (Marzo et al., 2015) developed a single-sided acoustic levitation array and does not require the reflector structure, which provides large operating area above the transducers array. This type of device can then be used to manipulate objects noninvasively in living body, like expelling a kidney stone (Ghanem et al., 2020) as shown in Figure 3A.

Transducer arrays can also be used to handle liquid using SAW, and have the advantage of being rewritable and programmable, compared with traditional acoustofluidic methods. In 2018, the team of Huang (Zhang et al., 2018) proposed an array of IDTs which can realize non-contact transportation of 1nL to 100 μL liquid along any planar axis via acoustic-streaming-induced traps. They also designed a hollow-square shaped IDTs and immersed it in water (Zhang et al., 2019), contactless and programmable manipulation of oil droplets and zebrafish larvae was realized on water, as shown in Figure 3B. Like PZT arrays, IDT transducer array can be arranged into other shapes than square matrix in order to accomplish complex two-dimensional patterning of single or multiple cells, such as fan-shaped (Wang et al., 2018) or ring-shaped (Tian et al., 2019) and this being very useful in the cultivation of special tissues. The phased array method for acoustic manipulation offers significant advantages in terms of real-time reconfiguration and precision. It allows for dynamic adjustments of acoustic waves, providing the flexibility to control ultrasound beams.

Acoustic holography introduces a simpler and more cost-effective approach compared to phased array transducers. It utilizes acoustic holograms to modify the output of a single ultrasonic transducer and create a designed 2D phase profile. The acoustic hologram plate could be fabricated with a costumed thickness distribution using the 3D printing technology, which is intend to form local phase retardation. This would modulate the phase shift physically (instead of electronically) to modify the phase shift in phased array. When ultrasound waves pass through the hologram, they emerge with the necessary phase distribution, diffract to form a real image at the desired location, and continue propagating. Therefore, the sound pressure distribution, designed to show high fidelity and independent of the host container geometry, is formed. Since the ultrasound is generated from one single transducer, the acoustic holography no longer requires a large number of transducers to achieve complex shaped manipulation. Melde et al. (Melde et al., 2016) were able to 3D print a hologram with 375 μm resolution and achieved 15,000 acoustic pixels in the hologram, where only one transducer operating at a frequency of 2 Mhz was used. This device can not only be used for acoustic fabrication of silicone particles soaked in a UV-crosslinker (Melde et al., 2018), but also used for building 3D cell assemblies (Ma et al., 2020a). Thus, acoustic technology has found various applications in the field of biomedical and biomaterials research. In the next section, some main biomedical applications of acoustic manipulation of micro/nano-motors and bioactive particles are reviewed.

## 3 Biomedical applications of acoustic driven micro/nano-motors and nanoparticles

### 3.1 Applications on cellular operations

**FIGURE 4:**
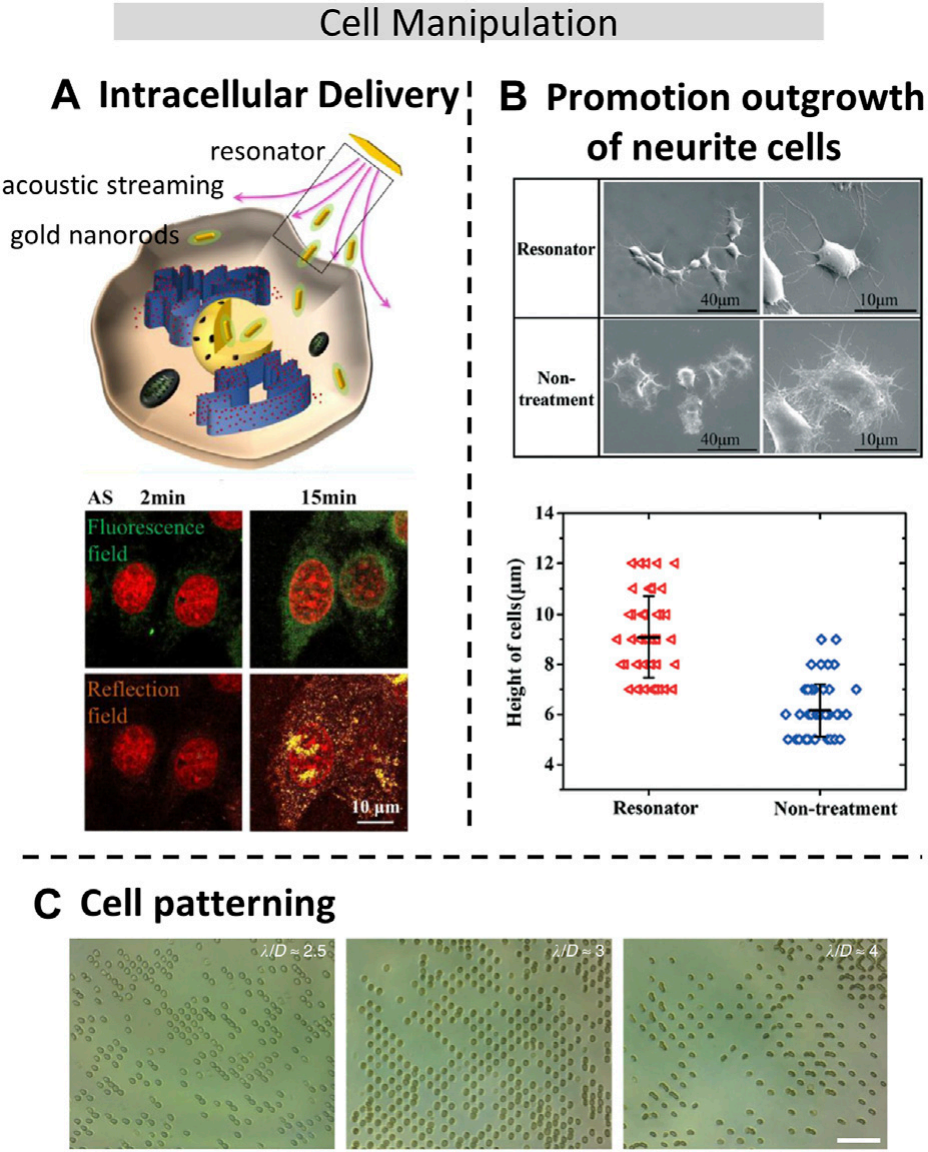
Acoustic manipulation techniques for cellular applications. **(A)**. Acoustic-induced intracellular delivery of gold nanorods (AuNRs) into the cytoplasm and even the nuclei of cancer and normal cells. Reproduced with permission from (He et al., 2022). Copyright 2022, American Chemical Society. **(B)**. The neuron-like phenotype of PC12 cells owing to shear stress caused by the acoustic streaming effect. Reproduced with permission from (He et al., 2021). Copyright 2017, The Royal Society of Chemistry. **(C)**. Red blood cell pattern in two dimensions using Standing Surface Acoustic Waves (SSAW) (Collins et al., 2015). Copyright 2015, The Authors. This work is licensed under a Creative Commons Attribution 4.0 International License.

Cell manipulation is a very important technology in bioengineering, micromanipulation has entered the operation level of subcellular level and has been applied to cell surgery, single-cell analysis, and cell translocation. Therefore, regulation of cell position and posture is an important part of cell micromanipulation. Among many non-contact cell manipulation techniques (Tang et al., 2022), both optical tweezers and electric methods could cause damage or induce undesired electrochemical reactions, while the acoustic methods could realize controlled rotation of biomaterials size ranging from 0.1 μm to 1000 μm. In addition to enabling cell rotation through the ultrasonic streaming flow effect, it also facilitates the transportation of complex trajectories (Ma et al., 2020b), or allows for controllable cell deformation (Guo et al., 2021) using bulk acoustic resonator. This study also shows that the cell deformation tool can be used for cell membrane permeability study, which could be used for research on cell surface modification and drug uptake efficiency. Zhao et al. (Zhao et al., 2021) developed a rapid drug screening method by changing the permeability of leukemia cells (THP-1), and increased the speed of drug screening by eight times. He et al. (He et al., 2022) have found that that acoustic treatment significantly facilitated intracellular delivery into both the cytoplasm and the nucleus, as shown in Figure 4A, with much higher efficiency compared to endocytosis. Additionally, they discovered that the acoustic treatment induced changes in the mechanical properties of both normal and cancer cells, leading to improvements in cytoskeleton integrity and cell stiffness. However, these effects were found to be dependent on the specific cell lines used in the experiments. When acoustic streaming is applied to nerve cells, it can also stimulate the neurite growth in the desired direction, as shown in Figure 4B (He et al., 2021). Compared to other methods, acoustic-based approaches are considered safer and more efficient. Another advantage of acoustic manipulation is the ability to cell patterning through the formation of standing waves, which is particularly useful in single-cell analysis. By adjusting the wavelength of the sound waves, the size of cell patterns can be changed to accommodate cells of different sizes. Unlike other cell manipulation techniques based on magnetic and electric field, acoustic manipulation does not impact cell viability, thereby preserving the cells’ biological activity and functionality. (Shi et al., 2009). achieved 1D and 2D cell patterning by placing the IDTs horizontally or vertically to form different SW acoustic fields. However, due to the larger wavelength of the acoustic waves compared to cell size, each trapping position was occupied by multiple cells. By increasing the frequency to reduce the wavelength to about one-fourth of the cell diameter, (Collins et al., 2015), realized single red blood cell pattern in two dimensions using SSAW, as shown in Figure 4C. This can also be used to the cultivation of special tissues such as engineering muscle tissue (Armstrong et al., 2018), cartilage (Armstrong et al., 2022) and guide neurite outgrowth (Gesellchen et al., 2014). Recently, (Zheng et al., 2023), assembled *in vitro* breast cancer cells (MCF-7) tumor spheroids by the use of acoustic microstreaming vortices generated with bulk acoustic waves. Biocompability is also crucial in cell manipulation (Wiklund, 2012), and it has been demonstrated that human white blood cells (Li et al., 2015), breast cancerous cells (Qian et al., 2021) can remain high viability after acoustic treatment.

### 3.2 Drug delivery and targeted therapy

**FIGURE 5:**
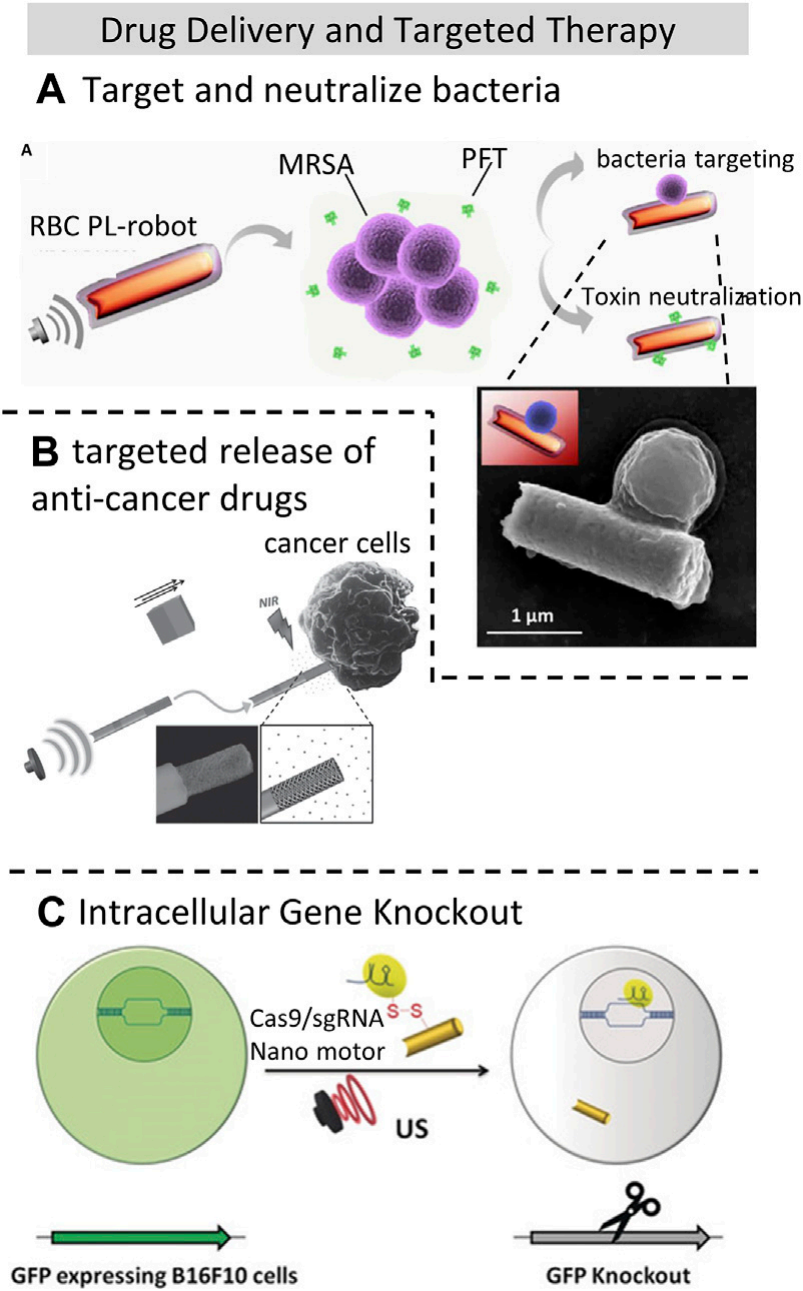
Drug delivery and targeted therapy applications using acoustic manipulation techniques **(A)**. Human red blood cells (RBCs) membrane coated nanorobots acoustically propelled to target and neutralize methicillin-resistant *Staphylococcus aureus* (MRSA) bacteria. Reproduced with permission from (Esteban-Fernández de Ávila et al., 2018). Copyright 2018, The Authors, some rights reserved; exclusive license American Association for the Advancement of Science. **(B)** Ultrasound-powered gold nanowire motors targeted release of anti-cancer drugs DOX to Hela cells. Reproduced with permission from (Garcia-Gradilla et al., 2014). Copyright 2014, WILEY-VCH Verlag GmbH and Co. KGaA, Weinheim. **(C)**. Co-delivery of paclitaxel and SsiRNA nanobubbles into lung cancer cells powered by Ultrasound. Reprinted (adapted) with permission from (Hansen-Bruhn et al., 2018). Copyright 2018 Wiley-VCH Verlag GmbH and Co. KGaA, Weinheim.

Micro/Nano-motors offer targeted drug delivery and release capabilities driven by external fields, where acoustically-driven micro/nano-motors have no limits to the materials used and thus offering higher safety and broader applicability than magnetically driven methods. Common types of ultrasonic motors include rod/wire-shaped, tubular, and helical micro/nanorobots (Li et al., 2022), where acoustically-driven gold nanowire robots are commonly used in medical applications. However, in the application of acoustic manipulation *in vivo*, challenges arise due to the varying acoustic impedance of different tissues, making precise guidance of medical motors to the target area difficult (Lee et al., 2020). There have been several studies combining drugs with micro/nano-motors for bacteria eradication and capture. For instance, acoustic-driven nano-motors (porous gold nanowire, p-AuNW) equipped with lysozyme can eliminate bacteria within minutes (Kiristi et al., 2015), benefitting from ultrasound propulsion, which efficiently removes dead bacteria adhered to the surface of the acoustic nano-motor. This has been demonstrated on M. lysodeikticus and *E. coli* bacterial models. Moreover, Esteban et al. (Esteban-Fernández de Ávila et al., 2018) successfully used acoustic propelled nanorobots to target and neutralize methicillin-resistant *Staphylococcus aureus* (MRSA) bacteria by employing gold nanowires cloaked with the membrane of human red blood cells (RBCs) as efficient toxin decoys, as shown in Figure 5A, where the trajectory of the nanorobots were recorded with a microscope. This has demonstrated the ability of acoustic propulsion to target and rapidly neutralize harmful pathogens.

Furthermore, acoustic-driven micro/nano-motors have also shown promise in targeted release of anti-cancer drugs (Garcia-Gradilla et al., 2014; Kiristi et al., 2015), as shown in Figure 5B. Wu et al. (Wu et al., 2015) utilized RBC-based micro-motors propelled by 2.4 MHz acoustic waves to deliver the chemotherapy drug DOX, reducing its toxicity to human umbilical vein endothelial cells (HUVECs). By comparison, the cell viability has decreased 19.7% by the use of free DOX. Encapsulating chemotherapy drugs in acoustic-propelled micro-motors can effectively reduce their toxicity to healthy cells, enhancing the effectiveness and safety of cancer therapy.

Additionally, ultrasound-driven nano-motors have enabled intracellular small interfering RNA (siRNA) delivery and gene silencing, for example, using an assembly of polymeric micelles and liposomes suppress the anti-apoptosis gene sirtuin 2 (SIRT2) in nude mouse glioma model (Yin et al., 2013) and the GFP (HEK293-GFP) gene-mRNA expression in living human embryonic kidney 293 cells using gold nanowires (AuNW) wrapped with a Rolling Circle Amplification (RCA) DNA strand (Esteban-Fernández de Ávila et al., 2016). The former study only utilized ultrasound to promote cell permeability, while the latter process was achieved through ultrasound-induced concentration of nano-motors and targeted cells into standing wave pressure nodes.

Moreover, (Hansen-Bruhn et al., 2018), effectively propelled Cas9/sgRNA-loaded gold nanowires (AuNWs) across the cell membrane, achieving efficient cleavage of the target GFP genomic sequence (95% after 48 h) with no significant impact on cell viability, as shown in Figure 5C. Furthermore, this team discovered that rapid acoustic propulsion of nano-motors, combined with the high oxygen loading capacity of red blood cell membrane-cloaked perfluorocarbon nanoemulsions (RBC-PFC), enables efficient oxygen delivery to the intracellular space of J774 macrophage cells and helps maintain their viability (Zhang et al., 2019). The acoustic field can accelerate intracellular oxygen delivery to cells, and the oxygen release rate can be adjusted by tuning the ultrasound intensity.

### 3.3 Acoustic-driven nano-motors for diagnostics

Acoustically-driven nano-motors can also be utilized for disease diagnosis. For instance, when these coated gold nanowires (AuNWs) are loaded with ssDNA that specifically binds to target mRNA (Esteban-Fernández de Ávila et al., 2015), which allows for the rapid detection of individual complete cells, enabling real-time disease monitoring for cancer diagnosis. It holds great potential in cancer diagnosis, patient follow-up, and monitoring disease progression. Similar methods have been applied for the detection of intracellular HPV16 E6 mRNA (Qualliotine et al., 2019), which is used for the diagnosis of Human papillomavirus (HPV)–associated oropharyngeal cancer (OPC) and the Alzheimer’s disease (Sun et al., 2021) using polystyrene microparticles as genetic biomarker carriers. In these methods, nano-motors are utilized to enter the cells through acoustic propulsion. They act as probes, binding to the target genes, which results in the switching of fluorescence signals. This switching of fluorescence signals enables the detection of specific genes. The use of these techniques allows for rapid and sensitive gene detection, providing powerful tools for biomedical research and diagnostics.

## 4 Discussion

Acoustic manipulation shows many advantages such as simplicity in fabrication, biocompatibility, non-contact operation, and compatibility compared with other microfluidic components. These features make acoustic manipulation suitable for applications in medical diagnosis and biochemical research. Despite the differences in the generation principles of BAW and SAW, the methods to control the position of sound traps during acoustic transportation and manipulation is remarkably similar. Acoustic manipulation not only enables cell rotation but also facilitates transportation along complex trajectories. Additionally, research shows that this technique can be used for studying cell membrane permeability, thereby enhancing drug uptake efficiency. Furthermore, improvements in gene silencing strategies were achieved through acoustically propelled nano particles, resulting in efficient, rapid, and accurate gene delivery and silencing response. This method holds great potential for gene therapy applications and serves as an efficient tool for targeted drug delivery. The review presents the progress of acoustic-driven micro/nanorobots in medical applications, providing novel insights for research, diagnosis, and treatment in related fields.

Applying acoustic manipulation techniques to *in vivo* operations stands as a significant challenge for the future. Within the internal environment, addressing the complexity of biological tissues, fluid dynamics effects, and precision of operations becomes crucial. Furthermore, exploring ways to enhance the manipulation capabilities of acoustically propelled micro/nanorobots, enabling more intricate cell operations and precise delivery of various types of drugs, emerges as a crucial research direction. The authors eagerly anticipate witnessing the evolution of this dynamic research field in the coming years, which is certainly worth further comprehensive exploration and dedicated investigation.
